# Metal wear-induced pseudotumour following an endoprosthetic knee replacement for Ewing sarcoma

**DOI:** 10.1007/s00256-017-2610-0

**Published:** 2017-03-07

**Authors:** Richard Craig, Marianna Vlychou, Catherine L. McCarthy, Christopher L. M. H. Gibbons, N. A. Athanasou

**Affiliations:** 1 Department of Orthopaedics, Nuffield Orthopaedic Centre, University of Oxford, Oxford, OX3 7HE UK; 2 Nuffield Department of Orthopaedics, Rheumatology and Musculoskeletal Sciences, Department of Histopathology, Nuffield Orthopaedic Centre, University of Oxford, Oxford, OX3 7HE UK; 3 Department of Radiology, Nuffield Orthopaedic Centre, University of Oxford, Oxford, OX3 7HE UK

**Keywords:** Endoprosthesis, Knee, Metal wear, Pseudotumour

## Abstract

Pseudotumours are well recognised as a complication of metal-on-metal hip arthroplasties and are thought to develop on the basis of an innate and adaptive immune response to cobalt-chrome (Co-Cr) wear particles. We report a case of a large pseudotumour that developed following a knee endoprosthetic replacement (EPR) undertaken for Ewing sarcoma. The lesion contained necrotic and degenerate connective tissue in which there were numerous scattered metal wear-containing macrophages, eosinophil polymorphs, lymphocytes, plasma cells and aseptic lymphocyte-dominated vascular-associated lesion-like lymphoid aggregates. Metal ion levels were elevated. No evidence of infection or tumour was noted and it was concluded that the lesion was most likely an inflammatory pseudotumour developing on the basis of an innate and adaptive immune response to components of Co-Cr metal wear derived from the knee EPR.

## Introduction

Fluid-filled or solid extra-capsular soft-tissue lesions, which have been termed pseudotumours, are a recognised complication of metal-on-metal (MoM) hip arthroplasties [1, 2]. These mass-like lesions form as a result of an innate and adaptive immune response to implant-derived cobalt-chrome (Co-Cr) wear particles [3–5]. The innate response is essentially a non-specific reaction to foreign material and, with regard to implants, is demonstrated largely as macrophage phagocytosis of wear particles. The adaptive immune response refers to the cellular response to a specific antigen, and in this context, refers to the cell-mediated (delayed hypersensitivity) reaction of primed T-lymphocytes to an antigenic wear particle-cell/tissue protein complex. Histologically, MoM pseudotumours exhibit a pronounced macrophage response to metal wear particles; these particles are cytotoxic and result in cell and tissue necrosis. In addition, pseudotumours often contain a pronounced lymphoid infiltrate that includes lymphocytes, plasma cells, occasional eosinophil polymorphs, and typically numerous perivascular lymphoid aggregates, a feature that has been termed aseptic lymphocyte-dominated vascular-associated lesion (ALVAL) [4, 6–10]; the lymphoid infiltrate reflects the adaptive immune response to metal wear particles.

Particle-associated lesions, some of which have been termed pseudotumours, have rarely been reported to develop in relation to a total knee arthroplasty (TKA) on the basis of a hypersensitivity response to polymer or metal arthroplasty components [5, 11–16]. Histological features of an adaptive immune response to metal and polymer wear components in periprosthetic tissues, similar to that seen in reaction to MoM hip implants, have been noted in failed knee arthroplasties [17, 18]. However, whether this reaction is associated with pseudotumour formation in the knee is uncertain.

We report an unusual case of a mass that developed after a knee endoprosthesis was inserted following distal femoral resection for a Ewing sarcoma. Clinical, radiological and pathological findings of this lesion, which showed morphological features of a necrotic and inflammatory response similar to that seen in MoM-hip related pseudotumours, are described and the role of hypersensitivity in the pathogenesis of knee implant failure discussed.

## Case report

**Fig. 1:**
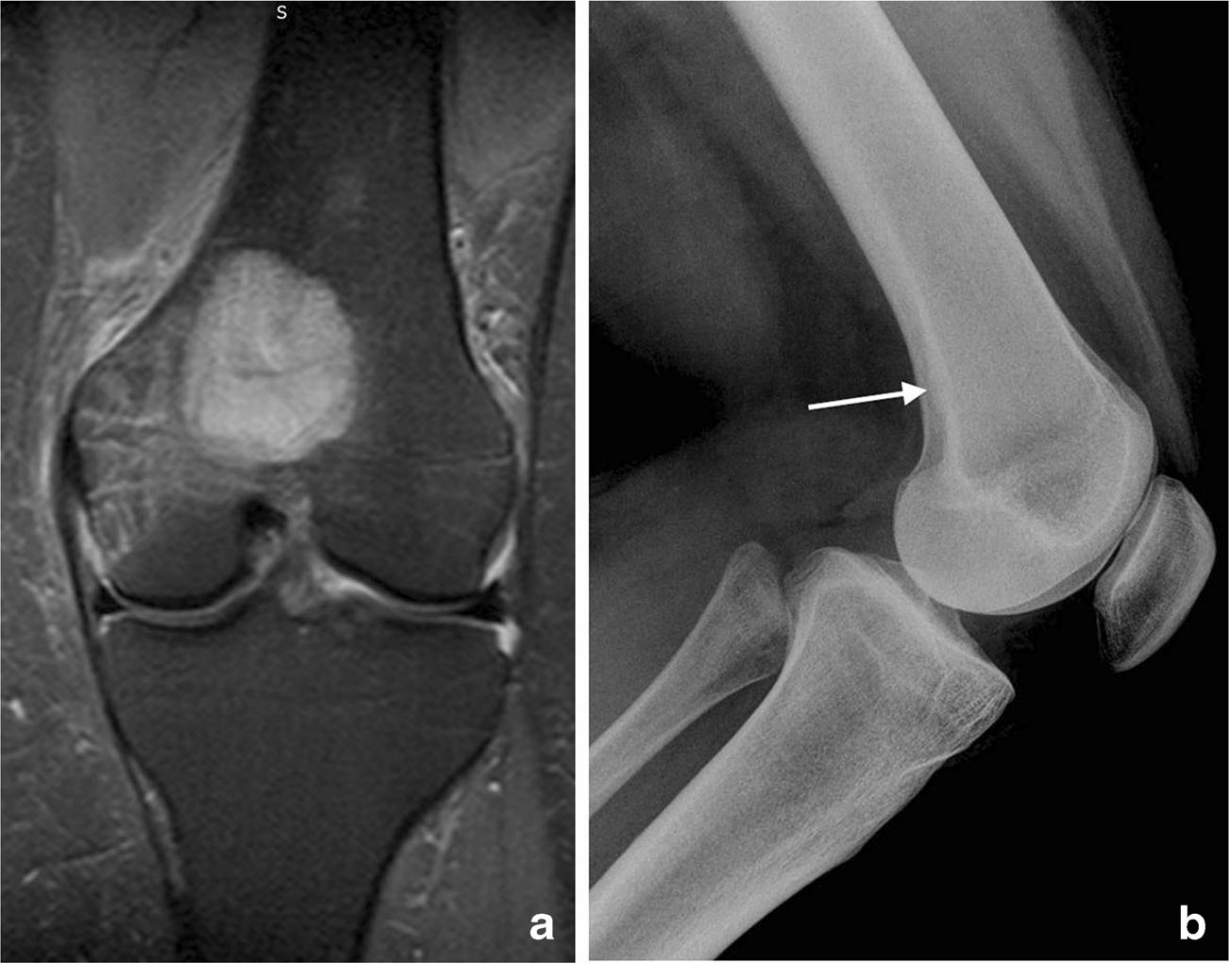
Imaging of the original tumour. **a** Coronal short tau inversion recovery (STIR) magnetic resonance (MR) image demonstrates a high signal lesion in the distal femoral metaphysis contacting the intercondylar notch and physis with adjacent bone marrow and soft-tissue oedema. **b** The lateral plain radiograph does not demonstrate the original lesion well, apart from posterior periosteal thickening (*arrow*). Histology was consistent with a Ewing sarcoma

**Fig. 2:**
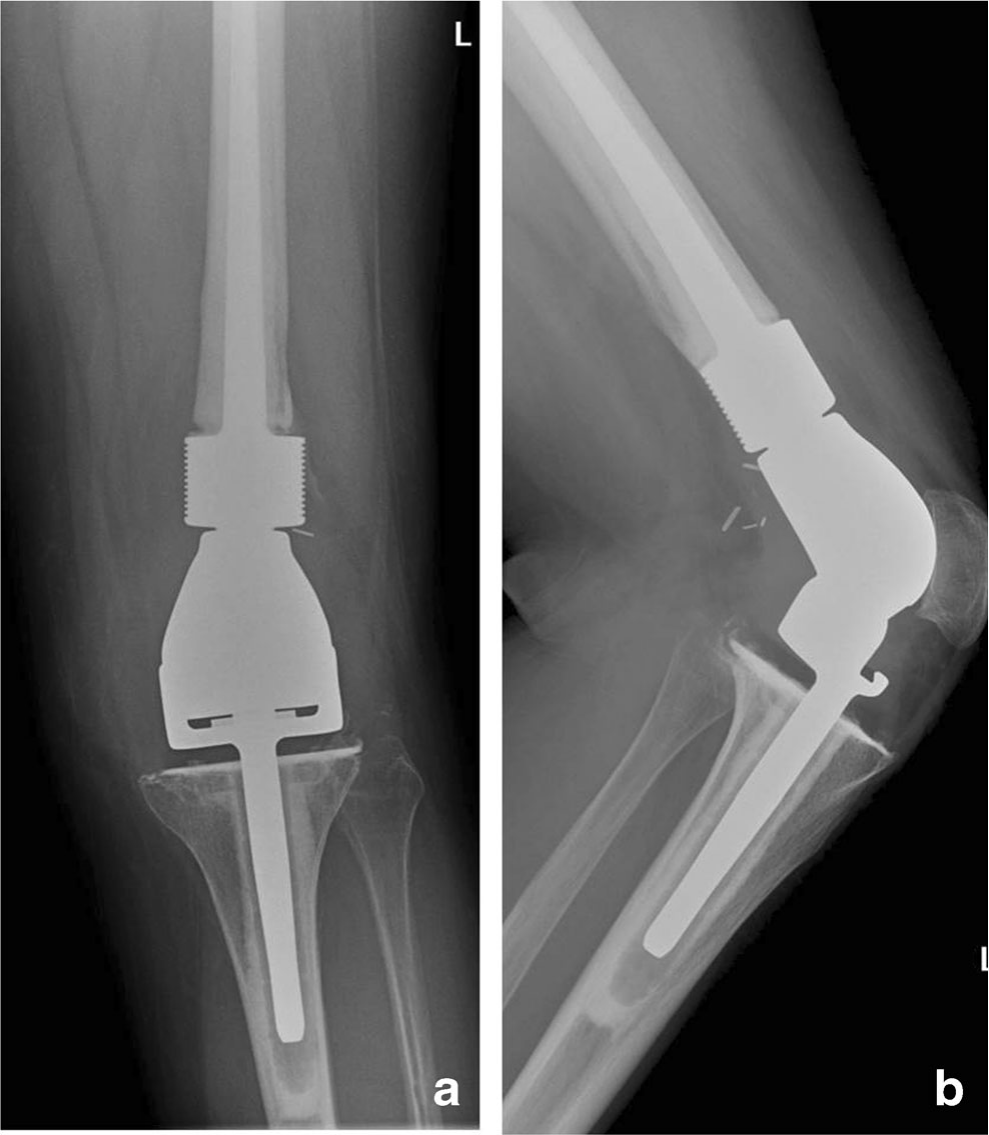
**a** Anteroposterior and **b** lateral plain radiographs post-resection of the distal femur and proximal tibia, with insertion of a distal femoral endoprosthesis, coupled to a rotating hinged tibial component

A 27-year-old female school teacher was diagnosed with a Ewing sarcoma of the left distal femur (Fig. 1). She was given six cycles of preoperative neoadjuvant VIDE vincristine, ifosfamide, doxorubicin, etoposide) chemotherapy before undergoing complete resection of the distal femur with insertion of a modular endoprosthesis (Fig. 2). The prosthesis was a METS modular cobalt-chromium-molybdenum (Co-Cr-Mo) distal femoral component with modular titanium shaft (Ti-6Al-4v alloy) and hydroxyapatite-coated collar, coupled to a Co-Cr-Mo metal-cased rotating hinged tibial component with ultra-high molecular weight polyethylene (UHMWP) bearings (Stanmore Implants Worldwide Limited, Elstree, UK). The resection was undertaken via an anterior extensile midline approach with medial para-patellar arthrotomy and complete excision of the tumour by resecting the distal third of the femur and proximal tibia with a normal cuff of tissue including genu articularis and vastus intermedius muscle. The rotating hinge of the EPR was assembled, inserted and coupled. This prosthesis is a rotating hinge with fixed femoral and tibial components. Attached by an axle is a rotating metal hinge within the tibial component and a plastic bumper to prevent MoM contact and wear.

Histological examination confirmed the diagnosis of Ewing sarcoma and noted that the tumour was fully excised. The specimen showed more than 90% necrosis of the tumour, indicating a good response to chemotherapy. No evidence of local or distant disease was seen on whole-limb MRI and whole-body PET scanning at follow-up. Postoperatively, the patient received adjuvant chemotherapy as part of the Euro Ewing protocol.

Six months after surgery, the patient developed anterolateral knee pain associated with mild patellar maltracking. Radiographs and clinical examination showed no abnormality. Her symptoms failed to improve and 1 year post-index surgery, she underwent exploration of the knee, biopsy and lateral release, which improved her patellar tracking. The synovium was swollen but otherwise showed no significant gross abnormality. Samples taken for microbiology showed no growth of organisms on culture. A period of comfortable normal knee function ensued.

**Fig. 3:**
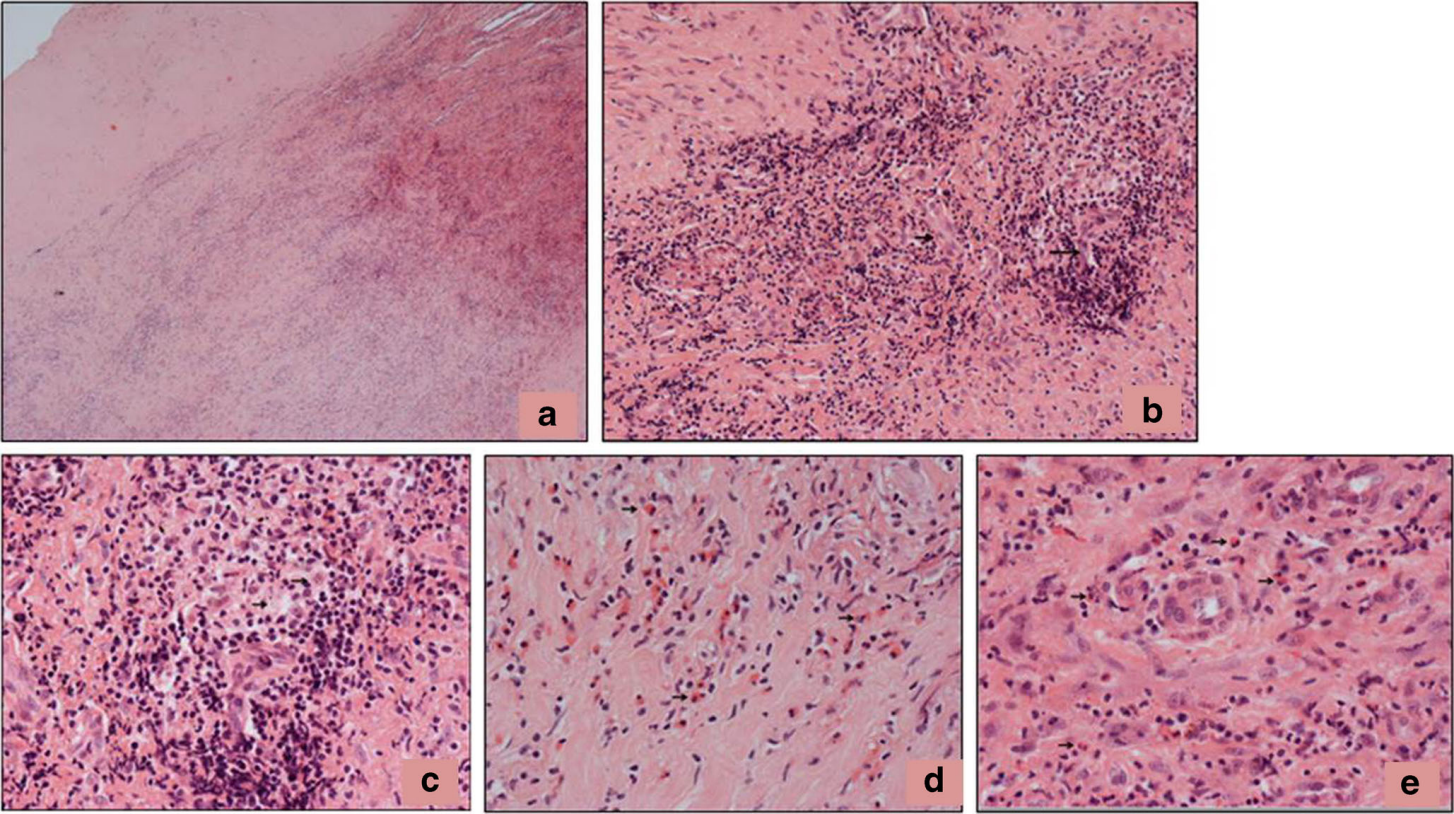
Histology of the capsule/synovium 42 months post-index surgery showing: **a** necrosis on the tissue surface with underlying diffuse inflammatory infiltrate; **b** focally heavy inflammatory infiltrate around small vessels lined by plump endothelial cells (*arrows*); **c** inflammatory infiltrate containing lymphocytes and macrophages, some of which contain black wear particles (*arrowed*); **d** prominent eosinophil polymorph infiltrate (*arrows*) with **e** evidence of vasculitis

Forty-two months following tumour resection, she presented with a swollen, painful knee, fever and night sweats. MRI showed considerable metal artefact, but no obvious evidence of recurrence. An ultrasound showed synovial thickening of the supra-patellar pouch, medial and lateral gutters, with no effusion. Her inflammatory indices were modestly raised (white cell count – 11 × 10^9^/L and CRP – 154 mg/L). Infection was again suspected and she underwent an open arthrotomy, arthrolysis and washout of the joint. Samples taken for microbiology showed no growth of organisms on culture. Samples of the capsule and synovium taken for histology showed extensive tissue necrosis and a heavy, diffuse inflammatory cell infiltrate that contained numerous eosinophil polymorphs, lymphocytes and macrophages (Fig. 3a). There was focal loss of the synovial lining; the surface of the synovium was covered in fibrin and necrotic tissue and there were necrotic and apoptotic macrophages. In deeper tissue, there was a heavy inflammatory cell infiltrate including numerous eosinophil polymorphs, lymphocytes and macrophages, some of which contained wear particles (Fig. 3b–e). There was evidence of vasculitis, with these inflammatory cells often concentrated around small vessels lined by plump endothelial cells (Fig. 3b–e). A few scattered neutrophil polymorphs were also noted within the inflammatory infiltrate. No evidence of tumour recurrence was seen and the histological findings were interpreted as showing features most in keeping with those of a hypersensitivity reaction, although it was noted that the possibility of infection could not entirely be excluded on these appearances. Following discussion by the multi-disciplinary team, it was decided that the patient should be treated with teicoplanin (600 mg for 6 weeks) through a central line and ciprofloxacin (500 mg bd) and rifampicin (300 mg bd) orally for 6 months. Her symptoms did not resolve with this treatment and 4 months later she underwent a repeat exploration and washout of the joint. Samples were sent for microbiology, but again showed no growth of organisms on culture.

**Fig. 4:**
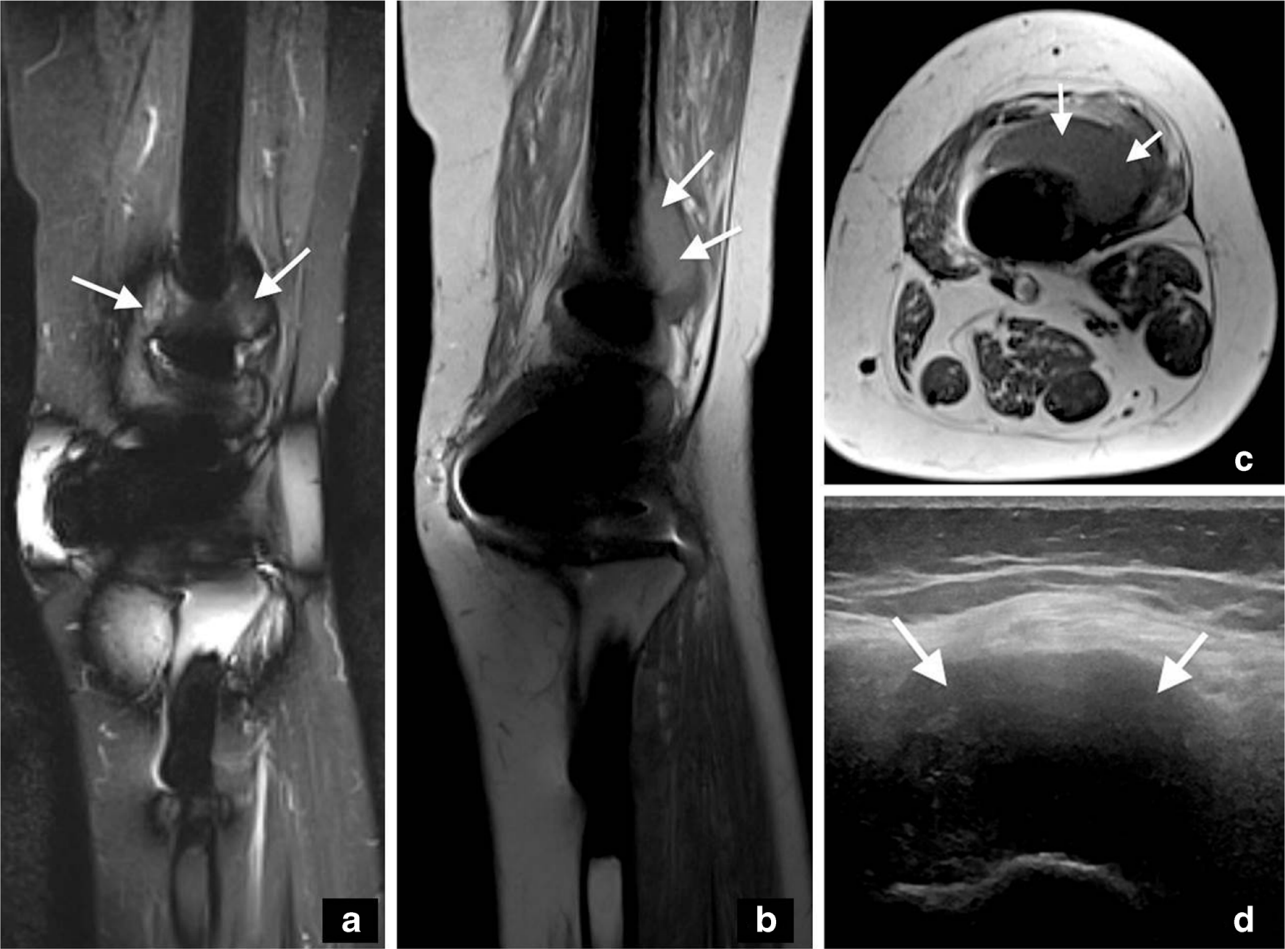
**a** Coronal STIR, **b** coronal T1, **c** axial T1-weighted MRI and **d** axial ultrasound image 6 years post-index surgery show a complex, predominantly thin-walled cystic soft-tissue mass intimately related to the antero-lateral aspect of the femoral component of the prosthesis (*arrows*). The lesion is well-defined, returns largely low STIR and is isointense to high T1-weighted signal relative to skeletal muscle, consistent with a pseudotumour. The diagnosis is supported by the homogeneous, hypoechoic appearance on ultrasound, with no Doppler activity

Her knee symptoms did not fully resolve after this treatment and 22 months later, she presented with a large mass around the knee. Ultrasound and MRI showed a complex, predominantly thin-walled cystic soft-tissue mass 7 × 6 × 5.5 cm encasing the implant. The mass had a homogeneous hypoechoic appearance at ultrasound with no Doppler activity, showed largely low signal intensity on short tau inversion recovery (STIR) imaging and was isointense to high T1-weighted signal relative to skeletal muscle on MRI (Fig. 4). She underwent an open biopsy, at which time it was noted that there was gross brown discolouration of the synovium/capsule (metallosis), but no evidence of loosening or component wear. Multiple tissue samples were sent for microbiology, but showed no growth on culture. Histology of the biopsy showed no definite evidence of tumour, but only necrotic tissue, in which there were scattered inflammatory cells, mainly macrophages and lymphocytes.

**Fig. 5:**
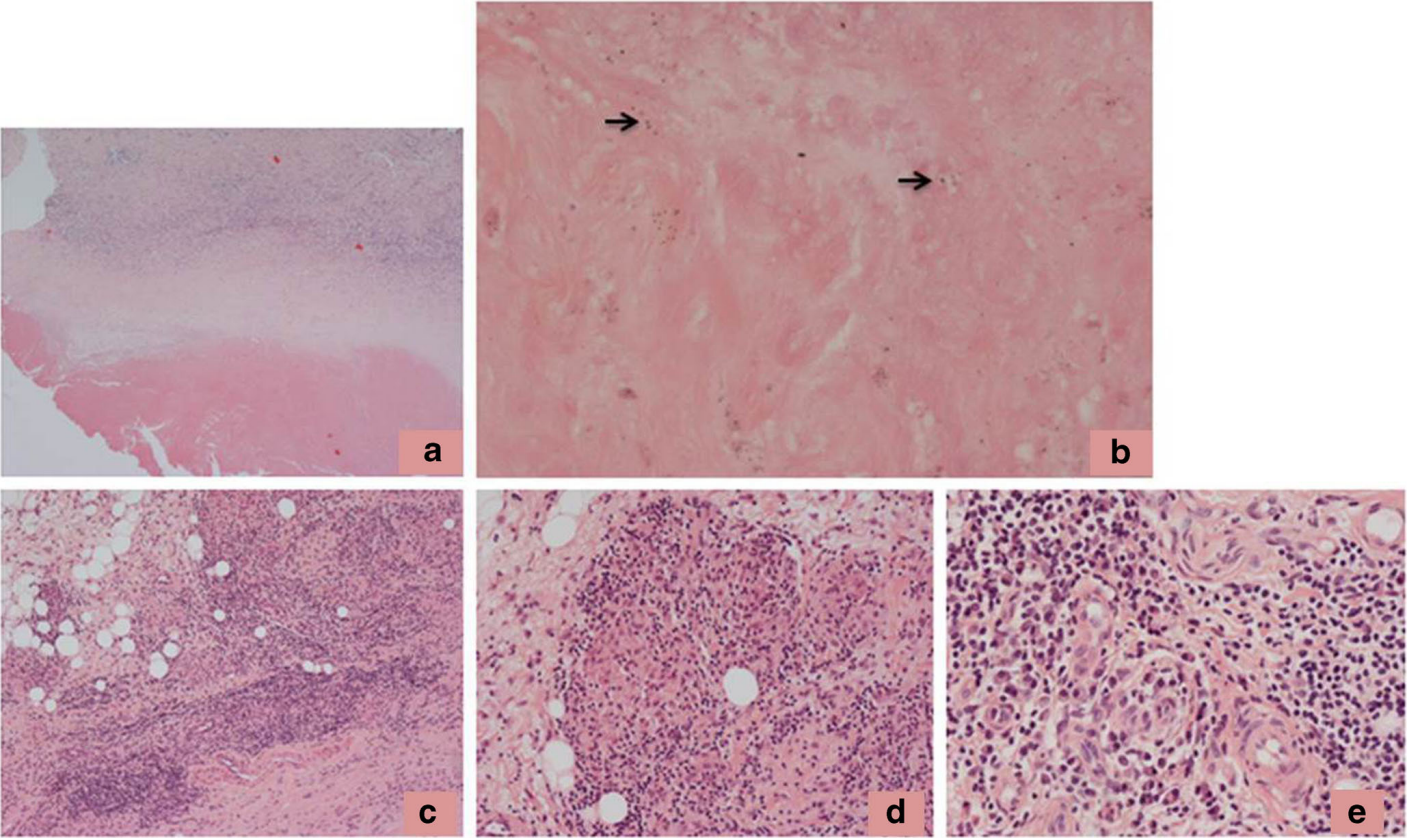
Histology of the pseudotumour 64 months post-index surgery showing: **a** characteristic zonal arrangement of the pseudotumour with a band of necrosis on the surface, an underlying zone of partly necrotic fibrous tissue containing macrophages, and deeper tissue containing a macrophage and lymphoid infiltrate; **b** necrotic macrophages and metal wear particles (some with* arrows*) in the superficial zone of the pseudotumour; **c** perivascular lymphoid infiltrate and scattered macrophages in deeper tissue; **d** granuloma-like collections of foreign body macrophages; **e** aggregates of lymphocytes and plasma cells around small vessels in the deeper tissue

A wide excision of the lesion was undertaken 4 weeks later. A prominent reactive synovitis around the implant was noted. The prosthesis was disassembled and a synovectomy and wide excision of the lesion undertaken with synovial and capsular excision anterior and posterior to the joint; this extended above the distal femoral component and femoral shaft. The implant was not revised. Samples sent for microbiology again showed no growth. Histology of the lesion showed a characteristic (MoM pseudotumour-like) zonal pattern with a central area of necrosis beneath which there was fibrous and granulation tissue containing numerous macrophages, and, in deeper tissues, a diffuse, focally heavy lymphoid infiltrate (Fig. 5a). The superficial zone contained abundant necrotic and degenerate connective tissue in which there was extensive deposition of metal wear particles, which showed the morphological characteristics of Co-Cr particles. No large birefringent particles, indicative of UHMWP wear, were seen, but it is possible that smaller (submicron) UHMWP particles, not visible on light microscopy, were present in the lesion. There were numerous necrotic and apoptotic macrophages containing wear particles (Fig. 5b). In deeper tissues, there was a diffuse, focally heavy, chronic inflammatory cell infiltrate, including numerous lymphocytes, plasma cells and macrophages (Fig. 5c). There were focal granuloma-like collections of macrophages (Fig. 5d) and there were prominent perivascular aggregates of lymphocytes and plasma cells (Fig. 5e). There was no marked eosinophil or neutrophil polymorph infiltrate. Immunohistochemistry showed that the lymphoid infiltrate was composed mainly of CD3+ cells. There was no evidence of recurrent tumour.

In light of the clinical history and the results of radiology, microbiology and other laboratory investigations, the histological findings were interpreted as showing features of an inflammatory pseudotumour that had arisen on the basis of an innate and adaptive immune response to components of metal wear derived from the knee EPR. Metal ion levels measured postoperatively were Co 64.5 nmol/L and Cr 32 nmol/L. These values were increased relative to the expected concentration for a metal on polyethylene hip replacement (Co and Cr 10 nmol/L and 15 nmol/L respectively).

Eleven months post-excision of the lesion, the patient remains clinically well, with no evidence of recurrence. As she is tumour-free 8 years after her initial surgery, her prognosis in this regard is excellent. She continues to have a swollen, painful knee and, although the implant components remain well-fixed, prognosis regarding her knee and the status of the prosthesis is guarded. She is being regularly followed up and her metal ion levels remain raised.

## Discussion

We report an unusual case of pseudotumour that developed several years after a knee EPR was inserted for Ewing sarcoma. Initially, the patient complained of recurrent knee pain and swelling. Microbiology investigations showed no evidence of infection and histology of the synovium taken 30 months after the knee EPR was inserted showed infiltration by inflammatory cells, tissue necrosis and features suggestive of vasculitis. She continued to complain of pain and intermittent swelling and 22 months later presented with a large mass around the knee. Tumour recurrence or infection was suspected clinically. Histology of the mass showed abundant necrotic tissue, numerous macrophages containing metal wear particles, and a heavy lymphoid infiltrate with numerous lymphocytes, scattered plasma cells and lymphoid aggregates concentrated around small vessels. Metal ion levels were raised and, on the basis that there was gross metallosis and that the inflammatory and necrotic features were similar to those seen in failed MoM hip implants, the mass was diagnosed as a metal wear-induced pseudotumour.

In keeping with this diagnosis, the ultrasound and MRI appearances of the lesion were similar to those of pseudotumours associated with MoM hip implant failure [19–21]. Pseudotumours can be complex with partly cystic and solid components. Typically, both the fluid contained in cystic lesions and the solid components of a metal-induced reactive mass show elements of low signal on STIR/T2- and intermediate to high signal on T1-weighted images reflecting the metal deposition [19]. Other causes of high T1-weighted signal, without gadolinium contrast medium, to consider in this setting include a collection containing proteinaceous fluid or methaemoglobin in a haematoma, but such lesions would be expected to return a corresponding high STIR/T2-weighted signal. Tumour recurrence was unlikely as the MRI signal characteristics did not correlate with the original tumour and ultrasound confirmed a predominantly cystic lesion with no internal vascularity.

To our knowledge, an inflammatory pseudotumour of this nature has not previously been reported to develop in association with a knee EPR inserted for surgical treatment of a malignant tumour. Approximately 12% of the volumetric weight of wear products of a TKA are metallic in origin, and concerns have been raised regarding the release of metal particles/ions from arthroplasty components, particularly in patients who may have metal hypersensitivity [20]. An increase in serum metal ion levels in paediatric patients following TKAs using megaprostheses has been noted and, on this basis, long-term follow-up has been recommended for such patients to identify and treat local and systemic effects of prolonged metal ion exposure [22]. Serum metal ion levels have been shown to be increased in loose TKAs in some studies [23, 24]. Cracchiolo and Revell [25] found that relative to normal controls, there was an increase in Co and Cr levels in synovial fluid from hinged TKAs with metal particles being noted in the fluid; however, they did not observe an increase in serum Co and Cr in hinged TKAs. In contrast, Luetzner et al. [26] noted an increase in both serums Co and Cr in well-functioning unconstrained TKAs relative to normal controls in which the reference values for both Co and Cr were < 0.25 μg/L. In our case, serum metal ions were significantly higher than this level, being 64.5 nmol/L (3.8 μg/L) for Co and 32 nmol/L (1.7 μg/L) for Cr, in keeping with metal wear playing a role in the pathogenesis of the inflammatory pseudotumour. Possible sources of the metal wear include the metal components of the rotating hinge and other exposed metal surfaces of the EPR.

The pseudotumours that form in relation to MoM hip arthroplasty develop as a result of an innate and adaptive immune response to components of implant-derived metal wear [3, 4]. Co-Cr wear particles and ions are known to be cytotoxic to macrophages, resulting in extensive tissue and cell necrosis. Elevated serum metal ion levels were noted in our patient, and raised serum and synovial metal ion levels have been reported in TKAs [22–27] A significant proportion of TKA patients are hypersensitive to metals found in implant components and metal hypersensitivity and metallosis plays a role some cases of TKA failure [5, 14–17, 28–30]. There have been reports of knee pain, swelling and dermatitis being associated with a hypersensitivity response to metal TKA wear components and some studies have concluded that hypersensitivity to metal implant components contributes to TKA implant failure [31–36].

Most reported cases of pseudotumour in relation to a TKA have been associated with the generation of abundant implant-derived polyethylene wear particles, which induce a heavy innate foreign body macrophage response and osteolysis [37–43]. There is usually little lymphocytic reaction in these metal-on-polyethylene pseudotumours, indicating that the adaptive immune response does not play a major role in their pathogenesis [44, 45]. In contrast, the pathological features seen in our case of pseudotumour showed features consistent with an adaptive (hypersensitivity) response with histology of the synovium and capsule initially showing numerous eosinophil polymorphs, macrophages and lymphocytes, and evidence of vasculitis; when the pseudotumour had developed, there was a diffuse, focally heavy lymphoid infiltrate, including ALVAL-like perivascular lymphoid aggregates [4, 7–9, 19]. A similar response is seen in skin lesions associated with contact dermatitis due to metal allergy; this can be induced by nickel, Cr and Co, all of which are present in knee implant components [3, 4]. Gross metallosis with numerous metal particles was seen in the pseudotumour that developed in our case. Metal ions can act as haptens, combining with larger carrier (cell or tissue-derived) proteins to become immunogenic. It is thus likely that, in addition to the known cytotoxic effect of metal ions, an adaptive (cell-mediated) hypersensitivity response to metal–protein complexes contributed to the necrosis and inflammation that led to pseudotumour formation in our case.

Our case illustrates that a pseudotumour can develop in relation to a knee EPR. This lesion needs to be distinguished from infection and recurrent tumour when the EPR has been inserted for surgical treatment of a bone/soft-tissue neoplasm. The pathogenesis of this lesion was most likely related to an innate and adaptive immune response to implant-derived components of metal wear as the clinical and pathological findings were essentially similar to those seen in cases of MoM hip implant-related pseudotumours. ALVAL-like perivascular lymphocytes and necrobiosis are commonly found in failed TKAs [16, 17], and this case provides some support for the concept of hypersensitivity being a possible contributory factor in TKA implant failure.
